# Changes in metabolic risk factors and gut microbiota during weight gain in male first‐year college athletes

**DOI:** 10.14814/phy2.70909

**Published:** 2026-05-10

**Authors:** Noriko Tanaka, Koichiro Hirano, Funa Kitagawa, Haruka Murakami, Hiroshi Akima

**Affiliations:** ^1^ Research Center of Health, Physical Fitness and Sports Nagoya University Nagoya Aichi Japan; ^2^ Graduate School of Education and Human Development Nagoya University Nagoya Aichi Japan; ^3^ Japan Society for the Promotion of Science (JSPS) Tokyo Japan; ^4^ Faculty of Sport and Health Science Ritsumeikan University Kusatsu Shiga Japan

**Keywords:** contact sports, fat accumulation, lipid metabolism, Shannon index, ultrasound imaging

## Abstract

Many contact sport athletes have high body mass index (BMI) and high metabolic disease risk. Rapid weight gain in first‐year athletes may further increase this risk and affect gastrointestinal status. This study examined metabolic risk factor and gut microbiota during weight gain in 21 first‐year male college athletes. We included members of student clubs for American football and rugby (ATH) and 10 controls without regular exercise habits (CON). Baseline measurements were taken within 3 months of joining the club, with follow‐up after 6 months. B‐mode ultrasound and blood and stool samples were analyzed. At the 6‐month follow‐up, the ATH group showed significant increases in body weight, weight‐adjusted thicknesses of subcutaneous fat and abdominal cavity, and triglyceride levels, while gut microbiota diversity significantly decreased. No significant differences were observed in the CON group. The rate of change in body weight in the ATH group was significantly positively correlated with the change in triglyceride levels (*r*_s_ = 0.436, *p* < 0.05) and significantly negatively correlated with the change in *Bacteroides* abundance (*r*_s_ = −0.631, *p* < 0.05). In conclusion, weight gain after joining athletic clubs in first‐year male college athletes was significantly associated with changes in blood lipid levels and gut microbiota.

## 1 INTRODUCTION

Contact sports such as American football and rugby involve intentional physical contact with opponents; therefore, athletes specializing in these sports tend to gain weight beyond the standard “normal” range to enhance their performance. However, cohort studies in Japanese people have reported that weight gain after reaching adulthood increases the prevalence of metabolic syndrome and diabetes in middle and later life (Takebe et al., 2021). It has also been suggested that, among Japanese male university students, those weighing 72.4 kg or more tend to have more fat mass than lean body mass (Takai et al., 2018). Excessive fat accumulation increases the risk of developing metabolic diseases. In fact, overweight athletes specializing in sports such as American football or judo exhibit a high prevalence of metabolic diseases, such as metabolic syndrome and insulin resistance, despite regular exercise habits (Borchers et al., 2009; Murata et al., 2016). Athletes who begin contact sports after entering university tend to gain weight through overeating as they are required to rapidly increase their body weight in a short period of time. This means that first‐year university athletes who have just started contact sports may be at increased risk of metabolic disease owing to this rapid weight gain. Weight gain can negatively affect future health.

Recent studies have reported that exercise participation is correlated with the profile of the gut microbiota and its metabolites. For example, the gut microbiota diversity in national level rugby players was significantly higher than that in healthy non‐athletes (Clarke et al., 2014). In contrast, rugby players at universities in Japan have lower gut microbiota diversity than healthy individuals without exercise habits (Morishima et al., 2021). This suggests that the diversity of the gut microbiota in athletes varies depending on their level of competition (Mohr et al., 2020). In addition, many athletes complain of gastrointestinal problems such as diarrhea during weight gain (Waterman & Kapur, 2012). Taken together, these results suggest that reductions in gut microbiota diversity may occur early in the weight gain process. However, little attention has been paid to changes in gut microbiota diversity during weight gain among first‐year university athletes.

This study aimed to clarify the changes in the risk factors of metabolic disease and gut microbiota over a 6‐month period following team enrollment among first‐year male college athletes participating in American football and/or rugby teams. We hypothesized that, after the observation period (6 months), freshmen would experience increases in subcutaneous and visceral fat levels, an increased incidence risk of metabolic diseases, and changes in gut bacterial diversity and dominance of specific bacterial species.

## 2 MATERIALS AND METHODS

### 2.1 Participants

We recruited first‐year students who were registered as members of athletic clubs in sports where body weight influences performance (American football, rugby, track and field throwing events, etc.) as well as students without regular exercise habits. Participants were recruited through flyers distributed at our and nearby universities, and through our university's subject recruitment system. Students who had used antibiotics or other antimicrobial agents within the preceding 2 months, who had chronic diseases or smoking habits, and/or who were unable to exercise for orthopedic reasons were excluded. In total, 22 students registered with American football and rugby clubs (ATH), and 10 sedentary students (CON) voluntarily participated in this study. One participant from the ATH group was excluded from all analyses because the gut microbiota data (*Bacteroides* genera abundance) at the 6‐month follow‐up exceeded than three standard deviations above the mean. Consequently, the final analyses included 21 students for ATH group (age: 18.7 ± 0.7 years, height: 174.4 ± 5.9 cm, body mass index [BMI]: 24.0 ± 2.6 kg/m^2^) and 10 sedentary students for CON group (age: 18.3 ± 0.5 years, height: 174.4 ± 5.8 cm, BMI: 20.1 ± 3.6 kg/m^2^). Among the ATH group, one participant (forward) was a member of a rugby club and 21 (linemen, tight ends, linebackers, running backs, and defensive backs) were members of an American football club. The teams belonged to either the first or second division of the Tokai Student League in Japan. Participants in the ATH group engaged in team activities 5 days per week, averaging approximately 3 h per day. Prior to the study, the participants were informed about the study's objectives, procedures, and potential risks, and written informed consent was obtained. This study was approved by the Research Ethics Committee of our university and was conducted in accordance with the Declaration of Helsinki. Regarding sample size, a power analysis was conducted for performing Mann–Whitney *U* test with an effect size of 1.25 (based on our unpublished data), a Type I error of 0.05, and a power of 0.80, the required sample sizes were 9 and 17 participants for each group, respectively. For the Wilcoxon signed‐rank test, assuming an effect size of 0.76 (based on our unpublished data and Stodden and Galitski (2010)), a Type I error of 0.05, and a statistical power of 0.80. The required sample size was 17 participants. For correlation analyses of change rates within the ATH group, assuming an effect size of 0.6 (Oliver et al., 2015), a Type I error of 0.05, and a power of 0.80, the required sample size was 21 participants.

### 2.2 Study protocol

The same measurements were taken twice for both the ATH and CON groups. Baseline measurements for the ATH group were taken within 3 months of joining the club (from May to July in 2022), and a 6‐month interval was set between the baseline and follow‐up measurements. Follow‐up measurement was conducted during the official game season namely, from November in 2022 to January in 2023. However, since participants in the ATH group were freshmen (non‐regular players), they were unable to participate in the official games.

### 2.3 Characteristics in body composition

Body weight, body fat percentage, and lean body mass were measured using a bioelectrical impedance method body composition analyzer (ITO‐InBody 370; Ito Ultrashort Wave Co., Ltd., Kawaguchi, Japan). The height and waist circumference at the umbilicus were also measured. Transverse images of five locations (anterior and posterior upper arm, abdomen, and anterior and posterior thigh) were acquired in the standing position using an ultrasound imaging device (Logiq e Pro; GE Healthcare JAPAN, Tokyo, JAPAN). The gain and measurement frequency were set to 45 dB and 10 MHz, respectively. Three images were acquired at each site. The anatomical locations of the measurement sites were as follows: anterior and posterior upper arm images were taken at 60% of the proximal distance from the acromion to the lateral epicondyle of the humerus; abdomen image was taken 2–3 cm below the umbilicus; and anterior and posterior thigh images were taken at 50% of the proximal distance from the greater trochanter to the knee joint space (Abe et al., 1994). Additionally, transverse images were acquired in the supine position 2 cm above the umbilicus for the measurement of abdominal cavity, an index of the accumulation of visceral adipose tissue (Stoner et al., 2015). During image acquisition of abdominal cavity, participants were instructed to inhale and hold their breath for approximately 5 s to minimize abdominal movements.

Using ImageJ software (ImageJ, National Institute of Health), thicknesses of skeletal muscle for the anterior and posterior upper arms, abdomen, and anterior and posterior thighs and subcutaneous fat above each skeletal muscle and the abdominal cavity were measured (Abe et al., 1994; Stoner et al., 2015). All three images acquired at each site were analyzed, the average thickness of each tissue was calculated, and the sum of these values was used for the analysis. Note that the thickness of each tissue is influenced by body weight; therefore, we adjusted thicknesses by their body weight^1/3^ (Kanehisa et al., 2004; Tanaka et al., 2025). This adjustment was based on the geometric scaling model, namely, MT is a one‐dimensional measure whereas body mass is a three‐dimensional one.

### 2.4 Metabolic risk factors

Blood samples were collected via finger pricks after an overnight fast using a blood test kit (Metabolic Syndrome & Lifestyle‐Related Disease Self‐Check; Approval No. for general medical devices: 22600BZX00362000; Demecal Co., Ltd., Osaka, Japan). Prior to blood sampling, participants were instructed to avoid eating after 8:00 pm the previous evening. Most participants collected their own blood immediately after waking up and before breakfast (between 7:00 am and 8:00 am). For the few participants who were anxious about self‐collection, blood was collected by the experimenter while they were fasting, immediately before the start of morphological and ultrasound imaging measurements (between 9:00 am and 10:00 am). Following collection, the sampling kit was promptly mailed to the testing center of Demecal Co., Ltd. The following blood parameters were assessed as indicators of metabolic disease risk: glucose, HbA1c, triglycerides, total cholesterol, high‐density lipoprotein (HDL) cholesterol, and low‐density lipoprotein (LDL) cholesterol. The measurements obtained using this kit have been shown to have a significant correlation (coefficient of determination *R* ^2^ = 0.955–0.986) with the measurements obtained from the median antecubital vein (Gootjes et al., 2009).

### 2.5 Gut microbiota

Stool samples were collected 0 to 2 days prior to body composition measurement using an intestinal microbiota testing kit (Mykinso Gut V4; Cykinso Co., Ltd., Tokyo, Japan) and stored at 4°C. Following body composition assessment, samples were mailed to Saikinso Co., Ltd., where subsequent analyses were performed. Stool collection, DNA extraction, base sequencing, and taxonomic identification were conducted according to previously described methods (Watanabe et al., 2021). Briefly, DNA was extracted from fecal samples using an automated DNA extraction system (GENE PREP STAR PI‐1200A, Kurabo Industries Ltd., Osaka, Japan) according to the manufacturer's protocol. The V1–V2 region of the 16S rRNA gene was amplified using forward primer (16S_27Fmod: TCG TCG GCA GCG TCA GAT GTG TAT AAG AGA CAG AGR GTT TGA TYM TGG CTC AG) and reverse primer (16S_338R: GTC TCG TGG GCT CGG AGA TGT GTA TAA GAG ACA GTG CTG CCT CCC GTA GGA GT) with KAPA HiFi Hot Start Ready Mix (Roche). To sequence 16S amplicons on the Illumina MiSeq platform, dual index adapters were attached using the Nextera XT Index Kit. Each library was diluted to 5 ng/μL, and equal volumes were pooled to a final concentration of 4 nM. The DNA concentration of the pooled library was quantified by qPCR using KAPA SYBR FAST qPCR Master mix (KK4601, KAPA Biosystems) using primer 1 (AAT GAT ACG GCG ACC ACC) and primer 2 (CAA GCA GAA GAC GGC ATA CGA). Library preparations were carried out according to 16S library preparation protocol of Illumina (Illumina, San Diego, CA, USA). Sequencing was performed using the MiSeq Reagent Kit v2 or v3 (500 or 600 Cycles) with 250‐ or 300‐bp paired‐end reads. Paired‐end reads of the partial 16S rRNA gene sequences were analyzed by using QIIME 2 (version 2020.8). The steps for data processing and assignment based on the QIIME 2 pipeline were as follows: (1) DADA2 for joining paired‐end reads, filtering, and denoising; (2) assigning taxonomic information to each ASV using naive bayes classifier in QIIME 2 classifier with the 16S gene of V1‐V2 region data of SILVA2 (version 138) to determine the identity and composition of the bacterial phyla and genera. Gut microbiota diversity and the relative abundance of gut bacteria at the phyla and genera level were measured using 16S rRNA microbiome analysis. Gut microbiota diversity was assessed using the Shannon index. For relative microbiota abundance at the genera level, analysis was performed on the top 10 genera with the highest abundance among the study participants.

### 2.6 Dietary assessment

Dietary habits during the previous month were assessed using a brief‐type self‐administered diet history questionnaire (BDHQ) for Japanese adults (Sasaki et al., 2000). Detailed information regarding the structure and calculation method of dietary intake by BDHQ was described elsewhere (Kobayashi et al., 2011; 2012). In this study, we focused on total energy intake and the percentage of energy derived from protein, fat, and carbohydrates.

### 2.7 Statistical analysis

The Shapiro–Wilk test revealed that some variables did not follow a normal distribution. Therefore, the median, 25th percentile, and 75th percentile values were used for all measurements. The Mann–Whitney *U* test was used to compare the ATH and CON groups at each measurement time point. The Wilcoxon signed‐rank test was used to compare the baseline and 6‐month values within each group. Factors associated with the percent changes (Δ) in characteristics of body composition, risk of metabolic disease, and gut microbiota were examined using Spearman's rank correlation coefficient. A significant threshold of < 5% was used for all analyses. SPSS Statistics (v20; IBM Corp., Armonk, NY, USA) was used for all analyses.

## 3 RESULTS

### 3.1 Characteristics of each group at baseline measurement

Characteristics of body composition at baseline are shown in Table 1. The ATH group showed significantly higher body weight, BMI, waist circumference, lean body mass, and skeletal muscle thickness than the CON group. No significant differences were observed between the groups in terms of body fat percentage, subcutaneous fat thickness, and abdominal cavity thickness.

**TABLE 1:** Changes in body composition.

Variables	CON (*n* = 10)						ATH (*n* = 21)					
	Baseline			6‐month follow‐up			Baseline			6‐month follow‐up		
	Median	25%	75%	Median	25%	75%	Median	25%	75%	Median	25%	75%
Body weight (kg)	56.4	49.4	66.2	58.2	53.3	62.5	74.4^a^	67.0	79.3	75.0^a^, ^b^	67.9	83.6
BMI (kg/m^2^)	19.1	17.3	22.2	19.8	17.9	22.1	24.4^a^	21.9	25.3	24.7^a^, ^b^	22.9	26.8
Waist circumference (cm)	69.4	64.4	79.2	71.0	67.8	75.4	82.1^a^	77.3	84.5	82.6^a^, ^b^	78.1	88.1
Body fat (%)	12.2	11.0	20.0	13.6	12.1	21.0	16.6	12.9	17.9	17.6^a^, ^b^	14.4	20.7
Lean body mass (kg)	48.4	43.2	53.8	48.3	44.3	52.6	59.6^a^	56.8	65.8	61.3^a^	56.2	68.6
Thickness/body weight^1/3^												
Skeletal muscle	44.2	42.4	45.2	45.1	42.4	46.2	49.0^a^	47.9	51.7	50.3^a^	48.6	52.5
Subcutaneous fat	7.2	5.3	11.1	7.8	5.0	13.2	7.3	6.3	10.7	8.1^b^	6.7	11.7
Abdominal cavity	11.0	10.2	12.4	11.6	10.9	12.4	13.8	11.4	15.2	14.0^b^	13.0	16.0

Abbreviation: BMI, body mass index.

^a^ Significantly different (*p* < 0.05) from CON at the same period.

^b^ Significantly different (*p* < 0.05) from baseline within same group.

Data on metabolic risk factors at baseline are shown in Table 2. No significant differences were observed between the groups for any measured parameter. Triglyceride levels exceeded the normal range (<150 mg/dL) (Committee to Evaluate Diagnostic Standards for Metabolic Syndrome, 2005) in 4.8% (*n* = 1) of the participants in the ATH group; all other values were within normal ranges in both groups. Note that since capillary blood reflects arterialized blood, blood glucose levels tend to be elevated (Priya et al., 2011). In fact, while the median blood glucose level in ATH was within the “high‐normal” range (Araki et al., 2020), no participants had impaired glucose metabolism in this study.

**TABLE 2:** Changes in blood properties.

Variables	CON (*n* = 10)						ATH (*n* = 21)					
	Baseline			6‐month follow‐up			Baseline			6‐month follow‐up		
	Median	25%	75%	Median	25%	75%	Median	25%	75%	Median	25%	75%
Glucose (mg/dl)	96.0	89.5	103.5	97.0	90.8	105.3	102.0	98.0	106.5	100.0	98.0	112.0
HbA1c (%)	5.2	5.1	5.3	5.3	5.1	5.5	5.2	5.2	5.3	5.4	5.3	5.4
Triglycerides (mg/dl)	50.0	43.8	68.5	48.5	37.5	58.5	77.0	56.0	85.3	92.0^a^, ^b^	70.5	134.0
Total cholesterol (mg/dl)	141.5	128.8	152.8	141.0	129.3	171.0	152.0	138.5	165.5	165.0	141.5	184.5
HDL cholesterol (mg/dl)	56.0	50.5	61.5	60.0	48.0	67.5	61.0	54.0	67.5	63.0	54.5	71.5
LDL cholesterol (mg/dl)	77.9	59.6	83.0	75.1	59.3	101.4	77.6	67.5	87.2	73.4	61.3	95.0

Abbreviations: HDL, high‐density lipoprotein; LDL, low density lipoprotein.

^a^ Significantly different (*p* < 0.05) from CON at the same period.

^b^ Significantly different (*p* < 0.05) from baseline within same group.

**FIGURE 1:**
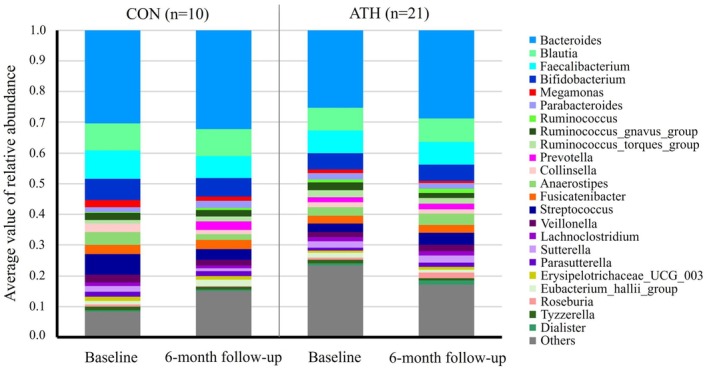
Averaged values of relative abundance of gut bacteria genera at baseline and 6‐month follow‐up measurements in each group.

Table 3 shows baseline data on the Shannon index, an indicator of the diversity of the intestinal microbiota, and the relative abundances of bacterial phyla and genera. Averaged values of relative abundance of gut bacteria genera at each time point were shown in Figure 1. Note that we compared the top 4 phyla and the top 10 genera. This was because the median for all participants was 0.0% and/or the overall abundance among participants was below 50%, making comparisons between groups difficult. As a result, a significant difference between groups was observed in the relative abundance of *Firmicutes* and *Bacteroidota* for the top 4 phyla. In contrast, no significant differences were observed between the groups for the top 10 bacterial genera.

**TABLE 3:** Changes in gut microbiota.

Variables	CON (*n* = 10)						ATH (*n* = 21)					
	Baseline			6‐month follow‐up			Baseline			6‐month follow‐up		
	Median	25%	75%	Median	25%	75%	Median	25%	75%	Median	25%	75%
Diversity of gut microbiota												
Shannon index	6.05	5.71	6.43	6.03	5.51	6.36	6.01	5.72	6.27	5.83^b^	5.43	6.10
Abundances of top 4 gut bacteria phyla (%)												
*Firmicutes*	58.81	52.85	65.12	47.46	45.67	49.61	52.8^a^	45.75	58.14	51.52^a^	48.63	55.80
*Bacteroidota*	23.56	16.91	32.66	41.93	35.22	45.44	35.5^a^	32.37	39.55	36.02^a^	30.77	40.71
*Actinobacteriota*	9.68	5.36	13.73	5.94	4.40	11.79	5.38	3.88	10.07	6.01	4.35	9.00
*Proteobacteria*	6.09	4.13	6.67	3.50	1.75	4.82	4.85	3.72	5.80	5.09	3.57	7.29
Abundances of top 10 gut bacteria genera (%)												
*Bacteroides*	31.3	26.1	35.4	33.9	26.3	38.7	28.1	20.2	30.4	30.5	23.9	35.3
*Blautia*	8.65	4.12	11.42	8.33	6.66	11.17	8.46	6.63	11.01	7.87	5.52	9.79
*Faecalibacterium*	4.63	1.38	10.92	8.84	2.92	11.24	8.43	7.26	12.52	8.87	5.71	10.58
*Streptococcus*	4.37	1.01	7.67	2.01	0.54	3.12	1.79	0.85	3.88	1.80	1.13	5.59
*Bifidobacterium*	4.08	2.10	7.43	5.01	2.20	8.59	4.54	2.75	8.22	3.95	2.55	7.00
*Fusicatenibacter*	2.13	0.98	4.67	2.56	1.30	4.31	2.24	1.42	3.77	2.85	1.07	4.17
*Sutterella*	1.81	0.00	3.62	0.04^b^	0.00	2.48	2.80	0.00	3.81	1.96	0.00	4.66
*Veillonella*	1.78	0.40	3.52	0.65	0.00	3.42	0.22	0.11	3.21	0.78	0.06	2.61
*Anaerostipes*	0.58	0.11	6.02	0.52	0.12	2.51	3.41	0.94	6.13	3.54^a^	1.05	4.94
*Prevotella*	0.00	0.00	0.02	0.00	0.00	0.00	0.01	0.00	0.09	0.00	0.00	0.01

^a^ Significantly different (*p* < 0.05) from CON at the same period.

^b^ Significantly different (*p* < 0.05) from baseline within same group.

In terms of dietary intake, total energy intake was significantly higher in the ATH group than in the CON group. There was no significant difference in other variables examined (Table 4). The results of intake of each food group are indicated in Table S1. Intake of cereals and fruits in ATH showed significantly higher values compared with CON. However, we should mention that there were participants who did not consume any food groups including fruits. Namely, 19.0% of ATH participants (*n* = 4) did not consume any food from a particular food group, resulting in data missing for 9.5%–14.3% within each food group. It makes it difficult for precise correlation analysis.

**TABLE 4:** Changes in dietary intakes.

Variables	CON (*n* = 10)						ATH (*n* = 21)					
	Baseline			6‐month follow‐up			Baseline			6‐month follow‐up		
	Median	25%	75%	Median	25%	75%	Median	25%	75%	Median	25%	75%
Total energy intake (kcal)	1689	1447	2360	1710	1518	2180	2752^a^	2346	3316	2722^a^	2246	3435
Protein (%)	14.0	12.5	16.3	13.0	12.1	16.1	13.4	11.5	15.1	13.1	12.0	13.6
Fat (%)	29.2	24.9	33.7	25.1	21.7	29.8	25.9	22.2	29.2	23.8	20.0	26.1
Carbohydrates (%)	56.1	52.4	60.9	61.3	53.2	66.0	60.4	58.2	66.3	62.9	60.2	68.1
Total dietary fiber (g)	8.2	6.8	11.4	8.1	7.1	13.1	11.0	8.6	13.7	12.7	9.5	16.1

^a^ Significantly different (*p* < 0.05) from CON at the same period.

### 3.2 Changes in each item over 6 months

The characteristics of body composition at 6‐month follow‐up are shown in Table 1. In the ATH group, body weight, BMI, waist circumference, body fat percentage, subcutaneous fat thickness, and abdominal cavity thickness were significantly higher at follow‐up than at baseline, with no significant changes observed in lean body mass or skeletal muscle thickness. Eleven participants (52.3%) of the athletes were classified as having overweight (BMI: 25.0 kg/m^2^ or higher (Committee to Evaluate Diagnostic Standards for Metabolic Syndrome, 2005)). No significant changes were observed in any parameters in the CON group.

In terms of metabolic risk factors in the ATH (Table 2), triglyceride levels were significantly higher at follow‐up than at baseline, with 14.3% (*n* = 3) of participants showing higher than normal values (normal: <150 mg/dL). However, no significant changes were observed in other items. In the CON group, no significant changes of metabolic risk factors were observed in any of the items.

In the ATH group, Shannon index values significantly decreased after 6 months, but no significant changes were observed in the abundance of individual phyla and genera (Table 3). In the CON group, the abundance of *Sutterella* significantly decreased after 6 months, but no significant changes were observed in Shannon index values or the abundance of phyla and other genera.

In terms of dietary intake, there was no significant change in any variables examined in the CON and ATH groups other than the dairy intake, which significantly decreased after 6 months in ATH (Table 4 and Table S1).

### 3.3 Factors associated with changes in body composition in the ATH group

ΔBody weight significantly associated with Δtriglycerides levels (*r*_s_ = 0.440, Table 5). There also existed significant relationships between Δskeletal muscle thickness and ΔLDL cholesterol (*r*_s_ = −0.461). No other significant associations were found among the variables examined in Table 5.

**TABLE 5:** Correlation coefficient between changes (%) in body composition and blood properties in ATH (*n* = 21).

Variables	ΔBody weight	ΔWaist circumference	ΔSkeletal muscle thickness/body weight^1/3^	ΔSubcutaneous fat thickness/body weight^1/3^	ΔAbdominal cavity thickness/body weight^1/3^
ΔGlucose	−0.105	−0.049	0.147	0.118	0.156
ΔHbA1c	−0.386	−0.167	−0.081	0.028	−0.298
ΔTriglycerides	0.440*	0.432	−0.100	−0.027	−0.099
ΔTotal cholesterol	−0.060	0.217	−0.419	0.157	0.096
ΔHDL cholesterol	0.116	−0.23	0.027	−0.120	0.247
ΔLDL cholesterol	−0.235	0.082	−0.461*	0.265	0.087

* *p* < 0.05.

In terms of gut microbiota (Table 6), Δbody weight showed a significant correlation with *ΔBacteroides* abundance (*r*_s_ = −0.631), and *ΔAnaerostipes* abundance (*r*_s_ = 0.640). ΔWaist circumference (*r*_s_ = −0.605) and Δabdominal cavity thickness/body weight^1/3^ (*r*_s_ = −0.447) also showed significant correlations with Δ*Bacteroides*. ΔSkeletal muscle thickness/body weight^1/3^ showed significant associations with Δ*Bifidobacterium* (*r*_s_ = 0.510) and Δ*Prevotella* (*r*_s_ = 0.449). No other significant associations were found among the variables examined in Table 6.

**TABLE 6:** Correlation coefficient between changes (%) in body composition and gut microbiota in ATH (*n* = 21).

Variables	ΔBody weight	ΔWaist circumference	ΔSkeletal muscle thickness/body weight^1/3^	ΔSubcutaneous fat thickness/body weight^1/3^	ΔAbdominal cavity thickness/body weight^1/3^
ΔDiversity of gut microbiota					
Shannon index	0.080	0.240	−0.251	0.304	0.070
ΔAbundances of top 4 gut bacteria phylm					
*Firmicutes*	−0.127	−0.018	−0.068	0.100	0.250
*Bacteroidota*	0.122	−0.048	−0.117	−0.318	−0.234
*Actinobacteriota*	0.205	0.288	0.270	0.350	0.090
*Proteobacteria*	−0.118	−0.091	0.120	−0.012	−0.192
ΔAbundances of top 10 gut bacteria genera					
*Bacteroides*	−0.631*	−0.605*	−0.142	−0.221	−0.447*
*Blautia*	0.106	0.278	−0.175	0.322	0.077
*Faecalibacterium*	0.318	0.410	−0.356	0.469	0.209
*Streptococcus*	0.038	0.126	0.143	0.084	0.384
*Bifidobacterium*	−0.064	−0.199	0.510*	−0.169	0.204
*Fusicatenibacter*	0.236	0.106	0.056	0.05	−0.142
*Sutterella*	0.037	−0.095	0.325	−0.366	0.367
*Veillonella*	0.042	0.087	0.022	0.149	0.322
*Anaerostipes*	0.640*	0.331	0.087	0.074	0.256
*Prevotella*	0.085	−0.065	0.449*	−0.295	0.261

* *p* < 0.05.

There was no significant correlation between variables in Δbody composition and Δdietary intakes examined (Table 7).

**TABLE 7:** Correlation coefficient between changes (%) in body composition and dietary intakes in ATH (*n* = 21).

Variables	ΔBody weight	ΔWaist circumference	ΔSkeletal muscle thickness/body weight^1/3^	ΔSubcutaneous fat thickness/body weight^1/3^	ΔAbdominal cavity thickness/body weight^1/3^
ΔTotal energy intake	0.155	0.199	−0.430	−0.136	−0.091
ΔProtein	−0.226	−0.408	0.192	−0.368	−0.055
ΔFat	0.108	−0.090	0.269	−0.281	−0.069
ΔCarbohydrates	0.003	0.213	−0.236	0.358	0.083
ΔTotal dietary fiber	0.181	0.29	−0.406	0.057	0.169

## 4 DISCUSSION

This study aimed to evaluate changes in metabolic risk factors and gut microbiota during weight gain in male first‐year university athletes belonging to the club of contact sports such as American football and rugby. The main findings of this study were as follows. (1) Compared to baseline measurements, body weight, BMI, waist circumference, body fat percentage, the body weight‐adjusted thickness of subcutaneous fat and abdominal cavity, and triglyceride levels were significantly higher at the 6‐month follow‐up in the ATH group, whereas no significant changes were observed in lean body mass or the bodyweight‐adjusted skeletal muscle thickness. (2) Compared to baseline measurements, gut microbiota diversity was significantly lower at the 6‐month follow‐up in the ATH group. (3) Changes in body weight showed a significant positive correlation with triglyceride levels and the abundance of *Bacteroides* bacteria. These results suggest the need for appropriate weight gain strategies for first‐year university athletes participating in the club of contact sports such as American football and rugby. As we hypothesized, short‐term weight gain in athletes is likely due to increases in subcutaneous and visceral fat rather than increases in skeletal muscle. This also indicates that appropriate weight gain strategies are necessary for first‐year university athletes.

Generally, BMI and body weight are considered as important determinants of gut microbiota composition. At baseline measurement, significant differences in BMI and body weight were already presented between the CON and ATH groups, likely because ATH participants may have begun gaining weight from the moment they decided to join American football or rugby clubs. One possible approach would have been to select a CON group with BMI values comparable to those of the ATH group (median: 24.7 kg/m^2^). However, several previous studies compared gut microbiota between groups with significantly different BMIs (e.g., Morishima et al. (2021)). Moreover, in Asian populations, a BMI of 25.0 kg/m^2^ or higher is commonly classified as obese or overweight, and a BMI close to 24.7 kg/m^2^ often indicates substantial accumulation of visceral and subcutaneous fat. Thus, selecting CON participants with similar BMI values may have resulted in an inappropriate comparison group for ATH. Importantly, at baseline, there was no significant difference in the thicknesses of subcutaneous and abdominal cavity, or genera‐level gut microbiota composition. Intergroup differences first emerged only at follow‐up measurement. Based on these findings, we considered that the differences in physique between the CON and ATH groups at baseline did not directly affect the findings of this study. Although significant differences in phyla‐level microbiota composition were observed at baseline and persisted at follow‐up (Table 3), the delayed emergence of genera‐level significant differences suggests that continued athletic training and associated weight gain may influence more specific microbial taxa beyond baseline compositional differences. Based on these findings, the differences in BMI between the two groups at baseline may not significantly affect the main outcomes of this study. Nevertheless, further research with larger sample sizes is warranted.

We hypothesized that weight gain associated with subcutaneous and visceral fat accumulation in male first‐year athletes would increase the risk of metabolic diseases. Metabolic syndrome is generally considered to develop due to the accumulation of visceral fat. Indeed, at the 6‐month follow‐up, 38.1% (*n* = 8) of participants in the ATH group exhibited a waist circumference of 85 cm or greater (a primary criterion in Japan's metabolic syndrome diagnostic criteria (Committee to Evaluate Diagnostic Standards for Metabolic Syndrome, 2005)). Although triglyceride levels changed significantly, no changes were observed in indices of glucose or lipid metabolism (Table 2). Japanese national‐level heavyweight judo athletes have significantly higher visceral fat mass and BMIs than heavyweight athletes in other sports; however, no significant differences in blood parameters were found when compared with non‐athletes (Murata et al., 2016). Furthermore, previous studies have shown that people with obesity with higher cardiopulmonary fitness have a lower prevalence of metabolic syndrome (Hong et al., 2014). The results of this study partially support these findings. However, the change in triglyceride levels showed a significant positive correlation with the change in body weight (Table 5). A significant negative correlation was also observed between changes in LDL cholesterol and changes in body weight‐adjusted skeletal muscle thickness. These findings suggest that not only overall weight gain but also changes in tissue distribution may affect lipid metabolism. Since most metabolic parameters remained within the normal range even after the 6‐month follow‐up, further research with dyslipidemia may be needed to clarify the mechanisms related to these relationships.

Shannon index values in the ATH group were significantly lower at the 6‐month follow‐up than at baseline (Table 3). The Shannon index is an indicator of the diversity of the gut microbiota, with high values indicating high bacterial diversity in the feces with an even distribution. The Shannon index is significantly lower in patients with allergies (Watts et al., 2021) or inflammatory diseases (Nishino et al., 2017) than in healthy individuals. The Shannon index is significantly negatively correlated with BMI (Liu et al., 2017), visceral fat mass (Ozato et al., 2019), and insulin resistance (Zhang et al., 2013). Additionally, Morishima et al. (2021) reported that Japanese university rugby players have lower gut microbiota diversity than healthy individuals without exercise habits. However, in the present study, the Shannon index did not significantly differ between the groups. Further, Δbody weight and Δabdominal cavity thickness did not show a significant association with the ΔShannon index (Table 6). The discrepancies between the findings of previous studies and those of the present study may be attributable to differences in participants' athletic history, competitive level, and the duration of the intervention or observation period. Notably, the Shannon index was significantly lower in the ATH group at the 6‐month follow‐up compared with baseline, suggesting that diversity of the gut microbiota may decrease in athletes who gain weight primarily driven by an increase in fat mass during their athletic careers.

The Δ*Bacteroides* genera abundance showed a significant negative correlation with Δbody weight and Δabdominal cavity thickness in the ATH group (Table 6). This suggests that a decrease in the abundance of *Bacteroides* is associated with weight gain and visceral fat accumulation. *Bacteroides* bacteria have high relative abundance in the intestines of Japanese individuals (Watanabe et al., 2021). These bacteria possess many genes that are involved in the degradation of polysaccharide‐containing dietary fiber, contributing to the production of short‐chain fatty acids (Tan et al., 2019). Additionally, the relative abundance of the *Bacteroides* genera has been shown to be significantly lower in people with obesity and type 2 diabetes than in healthy individuals (Andoh et al., 2016; Zhang et al., 2013). It has also been reported that individuals with higher levels of *Bacteroides* species exhibit lower levels of monosaccharides in the intestinal tract and a lower prevalence of insulin resistance (Takeuchi et al., 2023). Furthermore, monosaccharides such as glucose and fructose are thought to promote the production of inflammatory cytokines by immune cells in the body, thereby exacerbating insulin resistance and obesity (Dasu et al., 2008; Hannou et al., 2018). The administration of sodium alginate, a water‐soluble dietary fiber, to mice fed a high‐fat diet suppressed the onset of metabolic syndrome by increasing the number of *Bacteroides* species (Ejima et al., 2021). The present results support those of previous studies, suggesting that *Bacteroides* species may be involved in reducing the risk of metabolic syndrome.

The abundance of *ΔAnaerostipes* bacteria was significantly positively correlated with Δbody weight (Table 6). The *Anaerostipes* genera are characterized by their ability to produce butyric acid not only from dietary fiber but also from lactic and acetic acids (Bui et al., 2014). The concentration of butyric acid in the feces of patients with type 2 diabetes has been shown to be significantly lower than that in healthy individuals (Adachi et al., 2019). Furthermore, short‐chain fatty acids, including butyrate, may promote secretion of the gastrointestinal hormone GLP‐1 (Yadav et al., 2013). GLP‐1 is secreted by enteroendocrine cells in the intestine and promotes insulin secretion from the pancreas, thereby suppressing postprandial glucose levels (Rondas et al., 2013). Based on this, butyrate produced by butyrate‐producing bacteria such as members of the genera *Anaerostipes* may be involved in reducing the risk of metabolic diseases. However, there are reports indicating that butyrate concentrations in feces are significantly higher in people with obesity than those with normal body weight (Schwiertz et al., 2010). Further investigation is needed to interpret the results of this study, which showed a significant correlation in the ATH group between weight gain and the abundance of *Anaerostipes*.

This study has some limitations. First, the number of participants was relatively small, and the unequal group sizes may have limited statistical power. Second, the ATH group included participants from different sports (American football and rugby), playing positions, and training loads, however, these factors could not be fully accounted for in the analyses. Third, although dietary intake was assessed, we were unable to accurately quantify the intake of supplements and protein drinks. Fourth, since all participants were Asian men, the findings may not be generalizable to women or to individuals from other ethnic groups. Fifth, we compared the top 4 phyla and the top 10 genera only. We consider this analysis allowed us to assess the overall pattern of change during 6 months. Indeed, the top 10 genera accounted for over 70% of the total, and previous studies (e.g., Li et al. (2023)) also compared only the top 10 genera. However, it was true that changes also occurred in lower‐ranking genera. Next, since this was an exploratory study, we did not perform adjustments for multiple comparisons (e.g., false discovery rate correction) in correlation analyses. In fact, previous studies examining the association between changes in the gut microbiota and other variables, such as physical performance (e.g., Akazawa et al. (2023)), reported only unadjusted *p*‐values. However, false discovery rate correction is necessary in future studies. Finally, due to financial constraints, we did not evaluate microbial diversity other than Shannon index, i.e., β‐diversity, in the present study. Therefore, further investigations with larger and more diverse populations are required.

## 5 CONCLUSION

Although weight gain during the first 6 months of joining the club of contact sports such as American football or rugby was not particularly high, the body weight‐adjusted thicknesses of subcutaneous fat and visceral fat significantly increased, and significant changes in the indices of lipid metabolism and the gut microbiota diversity were observed. Nutritional interventions that begin immediately after joining such club teams may be necessary.

## AUTHOR CONTRIBUTIONS

**Noriko Tanaka:** Data curation; funding acquisition; investigation; methodology; project administration; supervision. **Koichiro Hirano:** Conceptualization; data curation; formal analysis; investigation; methodology. **Funa Kitagawa:** Data curation. **Haruka Murakami:** Supervision. **Hiroshi Akima:** Supervision.

## FUNDING INFORMATION

This work was supported by the personal research funds allocated by the corresponding author's affiliated institution and the RDP Program Aiming at Maximizing the Career Potential of Female Researchers, Nagoya University, (MEXT's Initiative for Realizing Diversity in the Research Environment, Leadership training type for women).

## CONFLICT OF INTEREST STATEMENT

There were no conflicts of interest in this study.

## ETHICS STATEMENT

This study was approved by the Research Ethics Committee of our university (21‐10).

## CONSENT

Prior to the study, the participants were informed about the study's objectives, procedures, and potential risks, and written informed consent was obtained.

## Supporting information


**Table S1:** Changes in dietary intakes of each food group.

## Data Availability

The data that support the findings of this study are available on request from the corresponding author. The data are not publicly available due to privacy or ethical restrictions.
