# Supplementary material for: Changes in metabolic risk factors and gut microbiota during weight gain in male first‐year college athletes
Source: Physiol Rep. 2026 May 10;14(9):e70909. doi: 10.14814/phy2.70909 (PMC13158145; doi:10.14814/phy2.70909)
Supplement: Supplementary file 1 — Table S1: Changes in dietary intakes of each food group. [file PHY2-14-e70909-s001.docx]

| Table S1. Changes in dietary intakes of each food group. | | | | | | | | | | | | | | | | |
| --- | --- | --- | --- | --- | --- | --- | --- | --- | --- | --- | --- | --- | --- | --- | --- | --- |
| Variables | CON (n=10) | | | | | | | | ATH (n=21) | | | | | | | |
|  | Baseline | | | | 6-month follow-up | | | | Baseline | | | | 6-month follow-up | | | |
|  | Median | 25% | 75% | non-consumer (%) | Median | 25% | 75% | non-consumer (%) | Median | 25% | 75% | non-consumer (%) | Median | 25% | 75% | non-consumer (%) |
| Food groups (g/day) | | | | | | | | | | | | | | | | |
| Cereals | 478.0 | 325.5 | 656.7 | 0.0 | 515.3 | 352.1 | 618.0 | 0.0 | 894.3* | 664.6 | 1046.7 | 0.0 | 945.5* | 669.0 | 1120.2 | 0.0 |
| Tubers | 42.7 | 22.5 | 95.3 | 0.0 | 52.0 | 24.8 | 79.4 | 9.5 | 25.4 | 11.9 | 57.7 | 0.0 | 25.4 | 10.8 | 67.8 | 14.3 |
| Sugars | 2.3 | 0.7 | 3.2 | 0.0 | 2.2 | 1.0 | 4.2 | 0.0 | 2.6 | 1.1 | 4.1 | 0.0 | 2.6 | 1.6 | 4.0 | 0.0 |
| Beans | 16.1 | 12.8 | 55.3 | 10.0 | 17.9 | 7.3 | 39.7 | 14.3 | 40.3 | 11.4 | 93.9 | 0.0 | 48.2 | 24.1 | 98.4 | 0.0 |
| Green and yellow vegetables | 65.4 | 26.6 | 150.7 | 10.0 | 92.7 | 53.4 | 113.2 | 0.0 | 115.2 | 97.6 | 138.2 | 0.0 | 88.5 | 65.9 | 176.5 | 0.0 |
| Light-colored vegetables | 111.3 | 77.8 | 155.4 | 0.0 | 95.0 | 66.6 | 138.5 | 0.0 | 120.5 | 87.4 | 167.8 | 0.0 | 126.2 | 90.9 | 176.0 | 0.0 |
| Fruits | 57.5 | 25.7 | 122.7 | 10.0 | 90.5 | 37.7 | 148.8 | 0.0 | 149.6* | 67.0 | 224.0 | 0.0 | 107.1 | 77.8 | 184.8 | 0.0 |
| Fishes | 33.5 | 6.9 | 86.1 | 0.0 | 31.9 | 11.3 | 51.3 | 4.8 | 37.6 | 21.8 | 64.4 | 0.0 | 51.4 | 30.8 | 81.1 | 0.0 |
| Meats | 120.9 | 55.4 | 146.7 | 0.0 | 103.6 | 65.5 | 144.0 | 0.0 | 149.2 | 112.3 | 181.9 | 0.0 | 104.5 | 84.4 | 189.8 | 0.0 |
| Eggs | 27.2 | 23.8 | 74.3 | 10.0 | 31.3 | 19.6 | 62.1 | 0.0 | 76.2 | 27.2 | 83.8 | 0.0 | 64.2 | 29.9 | 89.6 | 0.0 |
| Dairy | 108.3 | 70.4 | 195.4 | 0.0 | 119.1 | 39.7 | 164.7 | 4.8 | 227.7 | 126.8 | 398.4 | 9.5 | 87.2# | 22.7 | 225.7 | 9.5 |
| Fats | 17.6 | 13.1 | 19.8 | 0.0 | 12.5 | 10.7 | 16.8 | 0.0 | 19.8 | 13.1 | 25.8 | 0.0 | 17.7 | 11.8 | 22.3 | 0.0 |

*: significantly different (p<0.05) from CON at the same period, #: significantly different (p<0.05) from baseline within same group
